# Downregulation of Mitofusin 2 in Placenta Is Related to Preeclampsia

**DOI:** 10.1155/2016/6323086

**Published:** 2016-01-31

**Authors:** Jun Yu, Xijiao Guo, Ruibao Chen, Ling Feng

**Affiliations:** ^1^Department of Obstetrics and Gynecology, Tongji Hospital, Tongji Medical College, Huazhong University of Science and Technology, Wuhan 430030, China; ^2^Department of Urology, Tongji Hospital, Tongji Medical College, Huazhong University of Science and Technology, Wuhan 430030, China

## Abstract

*Background*. Mitofusin 2 (Mfn2) is a novel mitochondrial protein that is implicated in cellular proliferation and metabolism; however, the role of Mfn2 in preeclampsia (PE) remains unknown. This study aimed to explore the relationship between Mfn2 and PE.* Method*. Preeclamptic and normal pregnancies were enrolled in a comparative study. The expression of Mfn2 in placenta was detected by qRT-PCR. And the mitochondrial function was detected by ATP assay. Then TEV-1 cells were cultured in hypoxic conditions. mRNA and protein expressions of Mfn2 were detected by qRT-PCR and western blot separately. Cells' viability was detected by MTT. And the mitochondrial function was detected by ATP and mitochondrial membrane potential (MMP) assay. We further knocked down the Mfn2 gene in TEV-1 cells and evaluated the cells' viability.* Results*. Mfn2 and ATP expressions were significantly decreased in preeclamptic placentae compared to normal placentae. Mfn2 expression level and the viability of TEV-1 cells were reduced during hypoxic conditions. TEV-1 cells' viability, ATP, and MMP levels were also significantly decreased after knockdown of the Mfn2 gene.* Conclusions*. These results suggest that defects in Mfn2 could cause mitochondrial dysfunction and decrease trophoblastic cells' viability. Therefore, Mfn2 may be functionally involved in the pathogenesis of PE.

## 1. Introduction

PE is one of the leading causes of maternal and perinatal mortality and morbidity, which affects approximately 5–8% of all pregnancies, although there is a great degree of variation across regions [1]. The onset of a new episode of hypertension during pregnancy (with persistent diastolic blood pressure > 90 mm Hg) in conjunction with substantial proteinuria (>0.3 g/24 h) is generally used as the criteria for identifying PE [2].

To date, the etiology and pathogenic mechanisms of PE remain unclear. Increasing evidence suggests that mitochondrial defects play an important role in the initial stages of the pathogenic mechanisms leading to PE [3, 4]. Thus, some researchers have hypothesized that mitochondrial defects may cause the impairment of trophoblasts that leads to the severe placental disorder observed in PE [3–5]. This hypothesis provides potential new preventative strategies for PE. Evidence is accumulating that mitochondrial dysfunction is responsible for oxygen-sensitive accumulation and degradation, and impaired mitochondrial function is associated with PE syndrome [6, 7]. A series of studies have also identified alterations of mitochondrial proteins in the placentae of PE patients [3].

Mfn2 is a protein of the outer mitochondrial membrane that promotes membrane fusion and is involved in the maintenance of the mitochondrial network and bioenergetics. Mfn2 has a potential role in regulating cell proliferation and oxidative metabolism in many cell types [8]. Recently, Mfn2 has been reported to be an important biomarker and therapeutic target molecule for cardiovascular diseases such as hypertension [9, 10]. However, the functions of Mfn2 in the pathophysiology of PE remain undiscovered. This study was designed to investigate the correlation between Mfn2 and PE.

## 2. Materials and Methods

### 2.1. Patient Characteristics and Placenta Sampling

This study was approved by the Ethical Committee of Tongji Hospital, Tongji Medical College, Huazhong University of Science and Technology, China. All participants were informed that their placental tissue would be collected and that both their and their newborn's medical information pieces would be used once they were enrolled into this study. Their written consent was obtained before the initiation of the investigation. A case-control, cross-sectional, and comparative study was undertaken. Pregnant women with PE and pregnant women with normal pregnancies were included. All patients were consecutively admitted to each group. Inclusion criteria for both groups were uncomplicated singleton pregnancy, patient age between 20 and 35 years, and gestation > 33 weeks. Normal pregnancy was defined as a pregnancy in which the mother had normal blood pressure (less than 140/90 mm Hg), the absence of proteinuria, and the absence of medical or obstetrical complications. PE was defined according to the guidelines recommended by the American College of Obstetricians and Gynecologists (ACOG): onset of hypertension during midpregnancy or late pregnancy, with systolic and diastolic blood pressure equal to or higher than 140/90 mm Hg on at least two occasions that were separated by 6 h, and proteinuria of more than 300 mg in a 24 h period after 20 weeks of gestation [11]. The clinical characteristics of the patients are summarized in Table 1. All placental tissues were obtained immediately after delivery. The samples were first thoroughly washed with cold phosphate buffered saline (PBS), and then the villous tissues beneath the chorionic and basal plates were quickly dissected, sliced into small pieces (100–500 mg), snap-frozen in liquid nitrogen, and stored in −80°C freezer.

### 2.2. Cell Culture and Treatments

The immortalized TEV-1 cell line was obtained from normal extravillous trophoblast cells of a healthy first-trimester human placenta, which was a gift from the Chinese University of Hong Kong [12]. TEV-1 cells were grown in DMEM/F12 medium (Sigma-Aldrich, St. Louis, MO, USA) supplemented with 10% FBS containing 100 U/mL penicillin and 0.1 mg/mL streptomycin and incubated at 37°C with 5% CO_2_. To detect the effect of hypoxia on the expression of Mfn2 in trophoblastic cells, TEV-1 cells were treated with a hypoxic stimulus (CoCl_2_), as previously described [13]. TEV-1 cells were exposed to 300 *μ*M CoCl_2_ for 0, 12, 24, 48, or 72 h after being initially grown at 37°C for 24 h. Three replicated wells were used for each group. Following incubation, the cells were harvested for qRT-PCR, western blot, and MTT. HIF-1*α* level was used to evaluate the CoCl_2_-induced hypoxia. To explore the effect of Mfn2 on trophoblastic cell viability, TEV-1 cells were transfected with 50 nM of siRNA-Mfn2 or control oligonucleotides. Transfection was performed with Lipofectamine 2000 in OptiMEM (Invitrogen, Carlsbad, CA, USA) according to the manufacturer's instructions. Cell viability was assessed with transfected TEV-1 cells in 96-well plates using an MTT kit. The function of mitochondria was detected by ATP and MMP assay.

### 2.3. qRT-PCR Determination of Mfn2 Expression in Placental Villous Tissues

Total RNA was extracted using trizol reagent (Invitrogen, Carlsbad, CA, USA) as described previously [14]. The ratio of ultraviolet absorbance at 260/280 nm was determined as 1.8–2.0. RNA quality was detected by agarose gel electrophoresis. Those RNA, with their 28S : 18S being approximately 2 : 1, were subjected to reverse transcription using a kit (Toyobo Co., Ltd., Osaka, Japan) under the conditions, 30°C for 10 min, 42°C for 30 min, 99°C for 5 min, and 4°C for 5 min, and then stored at −20°C. qPCR was performed with an Mx3000P Real-Time PCR System using SYBR Green PCR mix (Toyobo Co. Ltd., Osaka, Japan) and the primer pairs, *β*-actin, 5′-GGGGTGTTGAAGGTCTCAAA-3′ (forward), and 5′-AGAAAATCTGGCACCACACC-3′ (reverse) and Mfn2, 5′-CCCCCTTGTCTTTATGCTGATGTT-3′ (forward), and 5′-TTTTGGGAGAGGTGTTGCTTATTTC-3′ (reverse). The reaction was carried out under the following conditions: 95°C for 2 min, followed by 40 cycles of denaturation at 95°C for 30 sec, annealing at 60°C for 30 sec, and extension at 72°C for 45 sec. The comparative threshold cycle (CT) method was used for relative quantification. All samples were performed in triplicate; data were averaged across the mRNA levels of each sample and then normalized to *β*-actin.

### 2.4. Cell Viability of Trophoblastic Cells in Hypoxic Conditions or Treated with siRNA

TEV-1 cells were seeded in 96-well plates at a density of 5 × 10^4^ cells per well and cultured with complete medium for initial 24 h for attachment. Then, cells were changed into fresh medium containing 300 *μ*M CoCl_2_ and cocultured for 0, 12, 24, 48, or 72 h. At the end of the time period, TEV-1 cell viability was tested by MTT assay as previously described [15]. Briefly, the treated cells were incubated with fresh media containing MTT for 4 h at 37°C in 5% CO_2_. Then, the supernatant was discarded, and 100 *μ*L DMSO was added to each well. Absorbance at 590 nm was measured on a multiwell microplate reader. The results are expressed as a percentage of the control.

### 2.5. Western Blot Assay of Mfn2 Expression

Cells were lysed in a lysis buffer at 0°C. The lysate was centrifuged at 16,000 g for 30 min, the supernatant was recovered and assayed for protein concentration, and a standard reference curve was obtained with the use of bovine serum albumin. Protein extracts (40 *μ*g per lane) were separated on a 10% SDS-PAGE gel and transferred onto PVDF membranes. Then, the membranes were subjected to immunoblot analysis as previously described [16]. Primary rabbit polyclonal anti-Mfn2, HIF-1*α*, and *β*-actin antibodies (Santa Cruz Biotechnology Inc., Santa Cruz, CA, USA; 1 : 1000) and secondary anti-rabbit horseradish peroxidase-linked whole antibody (Santa Cruz Biotechnology Inc., Santa Cruz, CA, USA; 1 : 5000) were used for these experiments. Antigen-antibody reactions were detected and visualized on film by chemiluminescence. *β*-actin was reprobed on the same membrane after washing as an internal control. The intensity of the Mfn2 and HIF-1*α* protein bands was quantified by Quantity One software and normalized to the amount of *β*-actin protein in the same sample.

### 2.6. Measurement of ATP

Direct measurements of ATP levels were obtained using an ATP assay kit according to the manufacturer's instructions (Beyotime, China). The kit is based on a luciferase-luciferin reaction assay. For these experiments, placental tissues and trophoblastic cells were split by the lysis reagent and subjected to centrifuge at 12,000 g for 5 min. 100 *μ*L of each supernatant was mixed with 100 *μ*L ATP working dilution. Luminance was measured using a monochromator microplate reader. The ATP levels were measured and the results were expressed as percentage of the treated control cells. For statistical analysis, the experiments were repeated three times.

### 2.7. Determination of MMP

Potential MMP was determined using the dual-emission mitochondrial dye JC-1 as described previously [17]. Cells were seeded in black-welled, 96-well plates, and the plate bases were covered before fluorescent reading. Cells were then incubated with JC-1 at a final concentration of 1 mM at 37°C for the last 30 min of the experiment. Red and green fluorescence were quantified using the fluorescent plate reader. The excitation peak was at 490 nm, with the emission maximum at 530 nm for the monomer, and the excitation peak and the emission maximum for the aggregate were 530 and 600 nm, respectively. The data were expressed as percentage of the treated control cells. And each experiment was repeated three times.

### 2.8. Statistical Analysis

All the statistical analysis was performed using SPSS 13.0 statistical software. Data are presented as the mean ± standard deviation. Nonparametric tests were used for skewed data and Student's *t*-test was used for normally distributed numerical variables. *p* < 0.05 was accepted as a statistically significant difference.

## 3. Results

### 3.1. Case-Control Study

Demographic characteristics from our case-control study are shown in Table 1. There were no differences in age, weight, or weeks of gestation among the groups. The body mass index (BMI) of the PE group was higher than the control group, and the difference was statistically significant.

### 3.2. Mfn2 and ATP Levels Were Significantly Decreased in the Placentae from the PE Group

**Figure 1:**
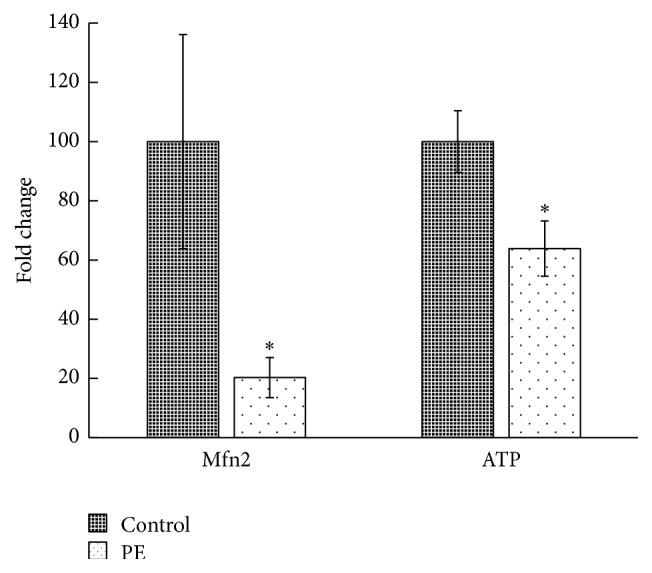
Mfn2 expression and ATP level in villous tissue. The expression of Mfn2 in placental villous tissue from both the control group and the PE group was assessed by qRT-PCR. The ATP level in placental villous tissue was assessed using a luciferase-luciferin reaction assay. The results showed that the Mfn2 mRNA expression and ATP levels in the villous tissue of the PE group were notably lower than in the control group (data are represented as the mean ± SD, ^*∗*^ *p* < 0.05).

The expression of Mfn2 in placental villous tissue was assessed by qRT-PCR. The ATP level in placental villous tissue was assessed using a luciferase-luciferin reaction assay. The results revealed that the Mfn2 mRNA expression and ATP levels in the villous tissue of the PE group were notably lower than in the control group (Figure 1). These findings suggest that expression of Mfn2 mRNA decreased in the placental villous tissues of patients with PE. And there is mitochondrial dysfunction in the placenta from PE patient.

### 3.3. Mfn2 Expression in TEV-1 Cells Was Reduced in Hypoxic Conditions

**Figure 2:**
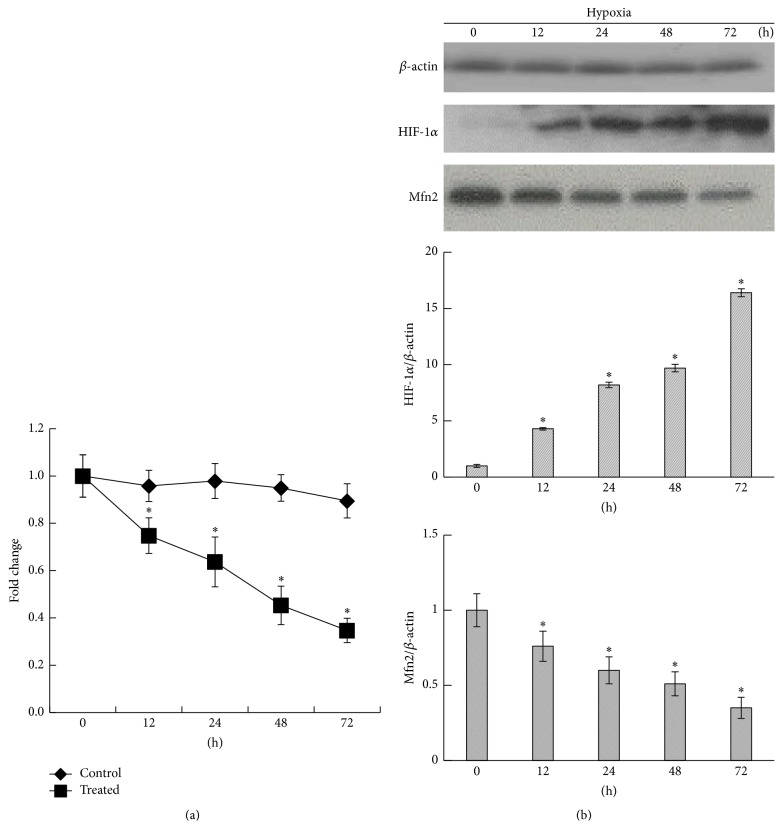
Mfn2 expression of trophoblastic cells in hypoxic conditions. TEV-1 cells were treated with 300 *μ*M CoCl_2_ for 0, 12, 24, 48, or 72 h. (a) Mfn2 mRNA levels in trophoblastic cells were determined using qRT-PCR. The results showed that CoCl_2_ treatment reduced Mfn2 mRNA levels in trophoblastic cells as early as 12 h after treatment, and the reduction was time-dependent (^*∗*^ *p* < 0.05). (b) The western blot results also showed that the Mfn2 protein level of trophoblastic cells decreased in a time-dependent manner when exposed to CoCl_2_, while the HIF-1*α* expression level increased (^*∗*^ *p* < 0.05). All data are presented as the mean ± SD of three independent experiments.

qRT-PCR analysis revealed that the Mfn2 mRNA levels were significantly reduced in TEV-1 cells exposed to CoCl_2_ as early as 12 h after exposure and decreased in a time-dependent manner (Figure 2(a)). The western blot results also showed that the Mfn2 expression of trophoblastic cells decreased in a time-dependent manner when exposed to CoCl_2_, while the HIF-1*α* expression level increased (Figure 2(b)).

### 3.4. TEV-1 Cell Viability Was Reduced in Hypoxic Conditions

**Figure 3:**
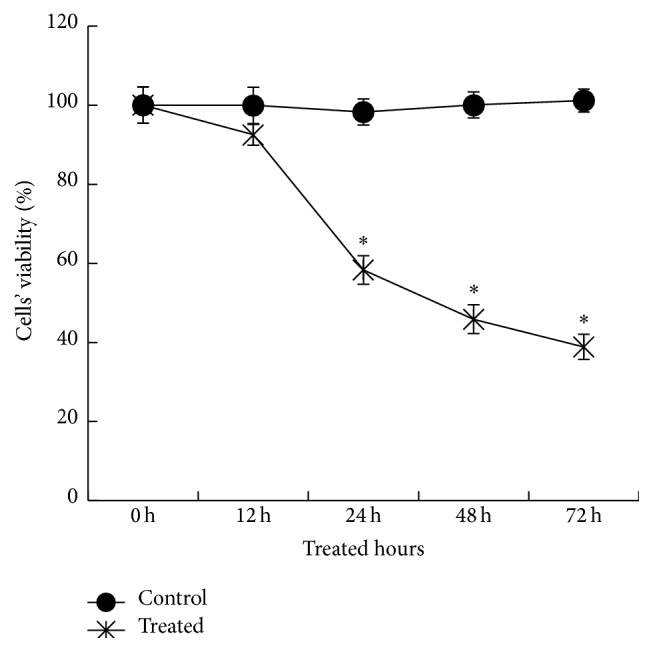
The viability of trophoblastic cells in hypoxic conditions. TEV-1 cells were treated with 300 *μ*M CoCl_2_ for 0, 12, 24, 48, or 72 h. Then, TEV-1 cell viability was determined by the MTT method. Data are presented as the mean ± SD of three independent experiments. The results showed that TEV-1 cell viability significantly decreased when cells were exposed to CoCl_2_, and the reduction was observed as early as 24 h after treatment. In addition, the reduction was time-dependent (^*∗*^ *p* < 0.05).

The MTT results showed that TEV-1 cell viability significantly decreased when exposed to CoCl_2_, and this decrease was observed as early as 24 h after treatment (Figure 3). TEV-1 cell viability also decreased in a time-dependent manner when exposed to CoCl_2_. The trend in TEV-1 cell viability in the hypoxic environment was similar to the trend in Mfn2 expression level, while the decrease of TEV-1 cell viability followed the decrease of Mfn2 expression level.

### 3.5. TEV-1 Cell Viability Decreased after Mfn2 Knockdown

**Figure 4:**
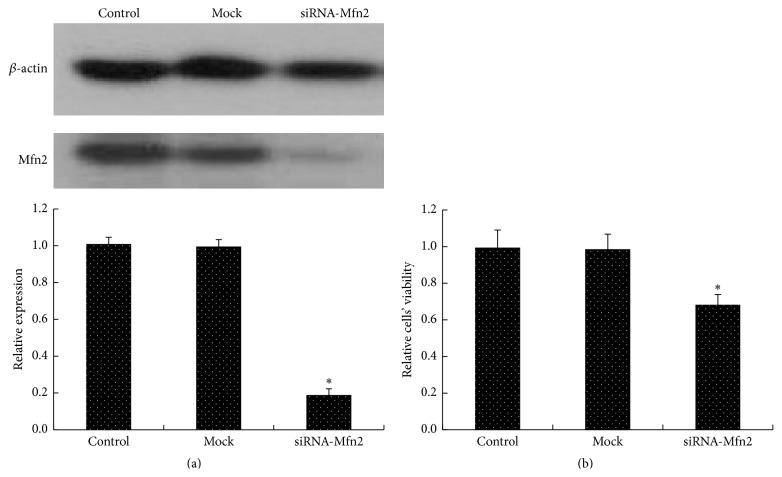
The viability of trophoblastic cells after Mfn2 knockdown. TEV-1 cells were transfected with 50 nM of siRNA-Mfn2 or control oligonucleotides. (a) The protein expression of Mfn2 in cells from the control group, mock group, and siRNA-Mfn2 group was detected by western blot. The results showed that the Mfn2 knockdown led to 81.33 ± 3.61% reduction of Mfn2 protein (^*∗*^ *p* < 0.05). (b) Trophoblastic cells from the control group, mock group, and siRNA-Mfn2 group were allowed to recover in full growth media for approximately 48 h before being incubated in low-mitogen media overnight. Then, cellular viability was assessed via an MTT assay. A significant loss of viability was observed with attenuation of Mfn2 (data are represented as the mean ± SD, ^*∗*^ *p* < 0.05).

To explore whether Mfn2 is integral to the survival of TEV-1 cells in culture, TEV-1 cells were treated with siRNA-Mfn2 or control oligonucleotides and allowed to recover in full growth media for approximately 48 hours; subsequently, cells were incubated in low-mitogen media overnight. Knockdown of Mfn2 led to 81.33 ± 3.61% reduction of Mfn2 protein (Figure 4(a)). Cellular viability was assessed via an MTT assay. Upon serum deprivation, a significant loss of viability was observed with the attenuation of Mfn2 (Figure 4(b)).

### 3.6. ATP Level and MMP in TEV-1 Cell Decreased after Mfn2 Knockdown

**Figure 5:**
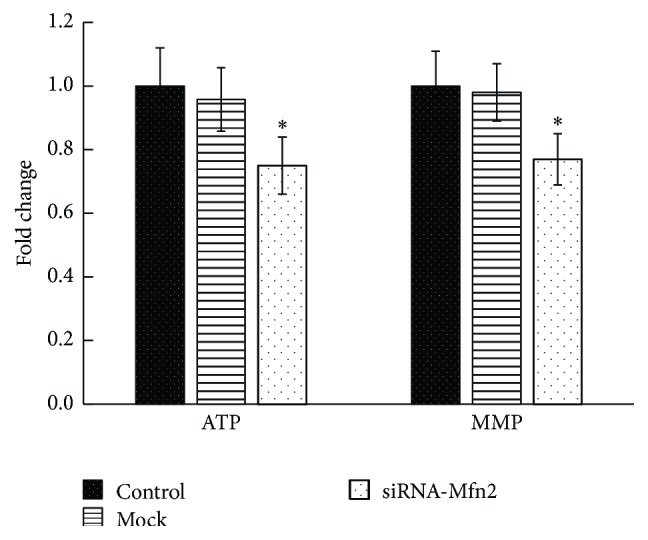
The ATP and MMP levels of trophoblastic cells after Mfn2 knockdown. The ATP and MMP were quantified based on fluorescence intensities. ATP level and MMP were both significantly decreased in siRNA-Mfn2 group, compared to the control group (^*∗*^ *p* < 0.05).

To explore mitochondrial function after Mfn2 knockdown, ATP level and MMP level were detected in TEV-1 cells treated with siRNA-Mfn2 or control. The results show that knockdown of Mfn2 led to significant reduction of both ATP level and MMP in trophoblastic cells (Figure 5).

## 4. Discussion

Placental dysfunction is the most common pathological change in pregnancy complications, which include PE. Recently, placental mitochondrial dysfunction has drawn the attention of researchers. Placental mitochondria provide most of the energy production in cells, participate in a number of important cellular processes, and are likely to play a central role in placental implantation, growth, and development. Some investigative teams have suggested that mitochondrial dysfunction may contribute to the pathogenesis of PE [3, 18]. However, there has been little research investigating the functional status of placental mitochondria in PE.

Mfn2 has been reported to be correlated with the pathological changes of some diseases related to mitochondrial signaling. In the present study, we found that the expression of Mfn2 in the placentae of the PE group was significantly decreased compared to the control group, which suggests that Mfn2 may be involved in the pathology of PE. We also measured the ATP, which represented key pathophysiological conditions in mitochondria [19]. Our results show that the placentae from PE patients possess a lower ATP level. These results confirmed the mitochondrial dysfunction in PE. PE is a pregnancy-specific disease characterized by the novel onset of hypertension and a series of other systematic disorders caused by renal injury (such as proteinuria and edema). Recent research demonstrates that Mfn2 could be an important biomarker and therapeutic target molecule for cardiovascular diseases such as hypertension. The expression of Mfn2 was markedly downregulated in vascular smooth muscle cells in spontaneously hypertensive rats [9]. Decreased expression of Mfn2 has also been found to contribute to mitochondrial fragmentation and a proliferation-cell death imbalance in human and experimental pulmonary arterial hypertension [10]. Other authors have also reported that Mfn2 deficiency exacerbates renal epithelial cell injury by promoting mitochondrial outer membrane injury and cell death [20]. Together with our results, these findings indicate that Mfn2 may be a potential biomarker and therapeutic target molecule for PE.

We further explored how Mfn2 expression in trophoblastic cells varies under hypoxic conditions. The results showed that the expression of Mfn2 mRNA in trophoblastic cells decreased in a time-dependent manner in the presence of a hypoxic stimulus, and the expression of Mfn2 was negatively correlated with HIF-1*α*. We also found that hypoxia significantly reduced TEV-1 cell viability. Both the Mfn2 expression level and the viability of TEV-1 cells were decreased in hypoxic conditions, although the decrease in Mfn2 expression level was observed prior to the decrease in TEV-1 cell viability. Previous studies have demonstrated that chorionic hypoxia can induce the trophoblast dysfunction and placental insufficiency syndromes of PE [21]. Our research shows negative relationship between Mfn2 and HIF-1*α*. These results combined the hypoxia-induced preeclampsia theory and mitochondrial dysfunction in PE. However, Mfn2 plays an important role in the pathogenesis of PE. Mfn2 is an important mitochondrial protein in the maintenance of mitochondrial bioenergetics and morphology. Recently, Mfn2 has been reported to have a potential role in regulating cell proliferation in many cell types [22, 23]. Mfn2 was found to have an important role in the activation of mitochondrial apoptosis pathways in cells; thus, the degradation of Mfn2 would lead to mitochondrial fragmentation and enhanced cell death [24]. A previous study also suggests that low Mfn2 expression is associated with apoptosis in the placental villi, which is consistent with our results [25]. Thus, our research could partially explain the decreased proliferation of trophoblasts, strengthening the hypothesis that Mfn2 plays a key role in trophoblast cell viability and acts as part of the molecular mechanisms underlying mitochondrial dysfunction in PE.

To confirm that Mfn2 is important for the survival of TEV-1 cells, we used siRNA to knock down the expression of Mfn2. To assess mitochondrial injury, we measured the MMP and ATP level. Our results indicated that Mfn2 knockdown leads to significant decrease of MMP and ATP levels. When Mfn2 expression was knocked down, cellular viability was also notably decreased. These results further indicated that the reduction of Mfn2 expression could reduce the proliferation of trophoblasts. Lugus et al. have reported that Mfn2 ablation diminishes endothelial cell viability and disrupts endothelial cell tube formation [26]. These results are consistent with our findings. Trophoblast cells exhibit high proliferation rates and play a crucial role in vascular remodeling of the placenta [27]. Any factors that lead to reduced trophoblast cell proliferation may result in poor vascular remodeling of the placenta, which is a key feature of PE.

Increasing evidence indicates that Mfn2 is strong regulator of metabolism. Mfn2 deficiency is associated with enhanced hydrogen peroxide concentrations, altered reactive oxygen species regulation, insulin resistance, and endoplasmic reticulum stress [28]. In this study, we also found that BMIs in the PE group were higher than in the control group. This result is consistent with previous reports [29]. However, the mechanism by which Mfn2 could regulate metabolism in the placenta needs further study. The results presented here provide evidence that Mfn2 may play a role in the multifactorial pathogenic mechanisms of PE.

Our research revealed that the level of Mfn2 in the PE group was notably lower than the level in the normal pregnancy group. This research also demonstrated that hypoxia decreased the expression of Mfn2 in trophoblastic cells, as well as cells' viability. And Mfn2 knockdown led to diminishing of trophoblastic cells' viability. Therefore, Mfn2 may be functionally involved in the pathogenesis of PE.

## Figures and Tables

**Table 1:** Perinatal characteristics of both study groups.

Characteristic	Control group (*n* = 16)	PE group (*n* = 16)	*p*
Weeks of gestation (w)	36.7 ± 1.96	36.4 ± 2.26	NS
Apgar (1 min)	7.9 ± 0.34	7.6 ± 0.50	NS
Apgar (5 min)	8.9 ± 0.25	8.5 ± 1.03	NS
Weight (g)	2923.9 ± 608.20	2470.0 ± 593.96	NS
Gravida	2.2 ± 1.5	1.8 ± 0.98	NS
Age (years)	26.7 ± 3.91	29.2 ± 4.78	NS
BMI	22.2 ± 9.36	28.5 ± 3.62	<0.05
Blood pressure			
Systolic	102.2 ± 11.27	148.2 ± 17.28	<0.05
Diastolic	72.4 ± 8.95	114.0 ± 10.73	<0.05

NS = not significant.
